# A Fusion-Based Technique With Hybrid Swarm Algorithm and Deep Learning for Biosignal Classification

**DOI:** 10.3389/fnhum.2022.895761

**Published:** 2022-06-03

**Authors:** Sunil Kumar Prabhakar, Harikumar Rajaguru, Chulho Kim, Dong-Ok Won

**Affiliations:** ^1^Department of Artificial Intelligence Convergence, Hallym University, Chuncheon, South Korea; ^2^Department of Electronics and Communication Engineering, Bannari Amman Institute of Technology, Sathyamangalam, India; ^3^Department of Neurology, Chuncheon Sacred Heart Hospital, Chuncheon, South Korea

**Keywords:** EEG, FHM, HDPAB, PMRM, deep learning

## Abstract

The vital data about the electrical activities of the brain are carried by the electroencephalography (EEG) signals. The recordings of the electrical activity of brain neurons in a rhythmic and spontaneous manner from the scalp surface are measured by EEG. One of the most important aspects in the field of neuroscience and neural engineering is EEG signal analysis, as it aids significantly in dealing with the commercial applications as well. To uncover the highly useful information for neural classification activities, EEG studies incorporated with machine learning provide good results. In this study, a Fusion Hybrid Model (FHM) with Singular Value Decomposition (SVD) Based Estimation of Robust Parameters is proposed for efficient feature extraction of the biosignals and to understand the essential information it has for analyzing the brain functionality. The essential features in terms of parameter components are extracted using the developed hybrid model, and a specialized hybrid swarm technique called Hybrid Differential Particle Artificial Bee (HDPAB) algorithm is proposed for feature selection. To make the EEG more practical and to be used in a plethora of applications, the robust classification of these signals is necessary thereby relying less on the trained professionals. Therefore, the classification is done initially using the proposed Zero Inflated Poisson Mixture Regression Model (ZIPMRM) and then it is also classified with a deep learning methodology, and the results are compared with other standard machine learning techniques. This proposed flow of methodology is validated on a few standard Biosignal datasets, and finally, a good classification accuracy of 98.79% is obtained for epileptic dataset and 98.35% is obtained for schizophrenia dataset.

## Introduction

A famous technique of assessing and measuring the electrical signals of the brain is done with the help of electroencephalography (EEG) (Jeong et al., 2020a). For the analysis of data concerned with both time and frequency domain, EEG is used and implemented as a powerful technique. The EEG can measure the voltage fluctuations arising due to the ionic current produced by the neurons of the brain (Jeong et al., 2020b). Over a period of time, the recording of the electrical activities across many scalp electrodes is done in a spontaneous manner to form an EEG signal. Cognitive behavior and major psychological activities can be easily traced by an EEG signal (Kwak and Lee, 2020), and various brain disorders can also be diagnosed and treated with the help of EEG signals such as stroke, epilepsy, sleep disorders, dementia, etc. (Lee et al., 2020a). EEG signals are also used for gaming purposes, to control objects using EEG monitoring, to manipulate different hardware utilizing brain waves, etc. (Marshall et al., 2013). The emotional transformation happening in the activities of the brain can also be specified by EEG (Lee et al., 2020b). The mechanisms which underlie the brain activity such as diagnosis of brain disease, human cognitive analysis, and brain computer interface (BCI) fields have gained a lot of attention too (Lee et al., 2020c). A simple operation principle coupled with a high-resolution time and low maintenance makes EEG more special than functional magnetic resonance imaging (fMRI) and computer tomography (CT) (Lee et al., 2019). Therefore, to study about the cognitive behavior in depth, to analyze the brain disorders, and to diagnose various mental disorders, the most famous non-intrusive approach is EEG. Machine learning and deep learning incorporated with EEG signal analysis is a very famous combination and has achieved wonders in the field of pattern recognition and soft computing (Suk et al., 2016). To improve the execution of a singular assignment, a collection of mathematical algorithms and models is used by machine learning. Training datasets are held as an input to be utilized as an escort for assembling estimates without the need for any distinct programming. Supervised and Unsupervised are the two main categories utilized in this space (Suk et al., 2015). With the help of machine learning methods, EEG signals are utilized as indicators to trace the specific medical conditions. From the EEG signal dataset, the noise and other outliers can be eliminated with the help of pre-processing. The spectrum of the grouping of data points to its corresponding features is expressed by feature extraction. Feature selection is utilized to select the most important features eliminating the redundant ones and finally the process of classification happens.

With respect to the discussion of previous studies and the necessity of this study, a few important and relevant studies with respect to epilepsy classification and schizophrenia classification from EEG signals are discussed below, as the proposed work in this study aims to classify them. Thousands of studies are available online in peer-reviewed journals and discussed in conferences for epilepsy classification from EEG signals, as this study has been under continuous development, modification, and improvement by various researchers at different instants of time. An overview to epilepsy and the revised classification of seizures was provided recently by Pack (2019). A comprehensive review on various pattern detection methodologies utilized for epilepsy seizure detection from EEG signals was reported by Sharmila and Geethanjali (2019). The applications of machine learning techniques for epileptic seizure detection and classification were analyzed by Siddiqui et al. (2020). Both these survey papers (Sharmila and Geethanjali, 2019; Siddiqui et al., 2020), published in 2019 and 2020, give enough information about the previously used methods, different techniques analyzed, various results, and its respective interpretations. An exhaustive review on the application of deep learning techniques for automated detection of epileptic seizures was beautifully analyzed by Shoeibi et al. (2020). Recent deep learning techniques, their implementation, datasets used, comparative analysis of results, and possible ways of future studies were thoroughly analyzed in the study. As far as the schizophrenia EEG signal classification is concerned, very few studies have made a commendable progress, and as it is an upcoming research field, only few studies are available in the literature. Some of the studies regarding schizophrenia EEG classification are mentioned as follows. A comprehensive information about the detection of schizophrenia using EEG signals was analyzed by Mahato et al. (2021). The classification of EEG signals between healthy and schizophrenia adolescents was done using fractal theory with approximate entropy analysis in Namazi et al. (2019) and Largest Lyapunov Exponents (LLE) was utilized with the help of Rosenstein algorithm for the EEG analysis of schizophrenia patients in Kutepov et al. (2020). The time and frequency domain features were combined with Convolutional Neural Network (CNN) and Long Short-Term Memory (LSTM) model reporting classification accuracies of 94.08 and 98.56% for healthy and schizophrenia patients, respectively (Singh et al., 2021). A deep convolutional network was designed where a classification accuracy of 98.07% was obtained for non-subject based testing and 81.26% for schizophrenia subject-based testing in Oh et al. (2019). A hybrid deep neural network which combines the usage of LSTM with CNN to classify the healthy vs. schizophrenia patients with fuzzy entropy features was reported with an accuracy of 99.22% (Sun et al., 2021). Deep learning methods with Random Forest based voting classifiers were efficiently used for the classification of schizophrenia EEG signals reporting an average accuracy of 96.7% (Chu et al., 2018). Nature-inspired algorithms with varied versions of Adaboost algorithm were performed for classification of schizophrenia EEG signals where they achieved a high classification accuracy of 98.77% (Prabhakar et al., 2020a). Similarly, several statistical feature analyses were made use with many swarm intelligence and optimization techniques, where Black Hole optimization technique with Support Vector Machine-Radial Basis Function (SVM-RBF) technique produced a classification accuracy of 92.17% in Prabhakar et al. (2020b). All the non-linear signal processing methods were comprehensively utilized for automated detection of schizophrenia reporting a classification accuracy of 92.91% with SVM (Jahmunah et al., 2019). An automated detection of schizophrenia using Empirical Mode Decomposition (EMD) and Intrinsic Mode Functions (IMFs) with several well-known classification methods produced a classification accuracy of 93.21% (Siuly et al., 2020), a multivariate iterative filtering technique was used for schizophrenia detection using SVM-cubic classifier reporting an accuracy of 98.9% (Das and Pachori, 2021), and finally, the concept of alpha band power during hyperventilation and post hyperventilation for the identification of schizophrenia was done reporting an accuracy of 83.33% (Bose et al., 2016). All the previous studies had equal merits and demerits depending on the algorithms and techniques employed to classify the signals. The main contributions of this study are mentioned as follows:

i) To the best of our knowledge, no one has ever proposed a fusion model with Singular Value Decomposition (SVD) and of robust parameters for the purpose of feature extraction of biomedical signals, and we have attempted and succeeded in it.
ii) The concept of a novel hybrid swarm algorithm was developed for the purpose of efficient feature selection, and it has performed well.
iii) A ZIPMRM is utilized for the biomedical signal classification, especially epilepsy classification and schizophrenia classification, and it is the first of its kind to do so and no past studies have reported it or made it available online.
iv) A deep learning methodology was also constructed for an efficient classification of the biomedical signals and the results are compared with the standard machine learning techniques.

**FIGURE 1:**
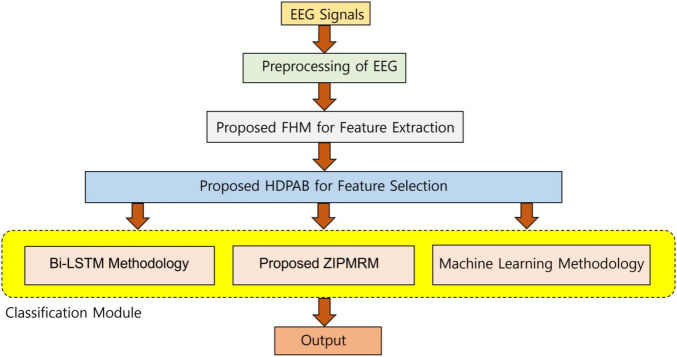
A very simplified workflow of the representation for an easy understanding.

The most important point to be observed in this research is the beautiful and novel convergence of the techniques such as the development and usage of the FHM for feature extraction, usage of a new hybrid swarm algorithm for feature selection, and utilization of ZIPMRM along with efficient deep learning methods for classification. The methods used in this study and the convergence of these techniques have not been reported in the literature so far and it is the first of its kind to implement it here. The simplified and structural flow of the study is expressed in Figure 1. The organization or the flow of the study is discussed as follows. The “Proposed fusion hybrid models (FHM) for feature extraction” section explains the concept of the proposed FHM for feature extraction and the “Proposed feature selection technique using HDPAB” section explains the concept of the proposed HDPAB algorithm for feature selection. The “Development of classification models” section gives the explanation about the usage of the proposed ZIPMRM along with suitable deep learning models for classification and it is followed by the results and discussion and finally conclusion.

## Proposed Fusion Hybrid Models for Feature Extraction

Ensemble models prove to be a great asset in the fields of statistics and machine learning models. A theoretical analysis and working of hybrid ensemble models were expressed by Hsu (2017). In different domains, ensemble learning has played a vital role such as visual tracking (Avidan, 2007), cancer classification (Cho and Won, 2007), email filtering (Katakis et al., 2010), intrusion detection (Govindarajan, 2014), fingerprint classification (Cappelli et al., 2002), protein food pattern recognition (Shen and Chou, 2006), and steganalysis (Kodovský et al., 2012). A review of ensemble techniques for bioinformatics was done by Pengyi et al. (2010). Other applications of fusion learning and modeling with machine learning include development of a hybrid data minimum model with feature selection algorithms (Koutanaei et al., 2015), development of a data-driven ensemble classifier (Hsieh and HungA, 2010), detection of iron ore sintering characters (Wang et al., 2019), and detecting the temperature of molten steel in ladle furnace (Tian and Mao, 2010). The recent facilitation in machine learning representation assessing hybrid and ensemble methods was reported by Ardabili et al. (2020). However, in the literature, such fusion models are not available for the purpose of feature extraction from biomedical signals and so this motivated the researchers to undertake this study.

For various types of data, the specific proposal of different mixture model techniques has been developed. The merits of various techniques have been integrated into one package. The specific model which fits the data in the best possible manner is done by this fusion approach and it is highly dependent on the data that have to be analyzed. For the fusion model developed here, the initial mixture model considered is the gamma-normal-gamma (GNG) model (Dean and Raftery, 2005; Bhunya et al., 2007). Multiple normal components are utilized in order to capture the data, and therefore, a unique case of gamma distribution is utilized. GNG was alter integrated with uniform-normal mixture model (NUDGE), which utilizes a single normal component and a single uniform component (Dean and Raftery, 2005). The NUDGE was later extended and termed as eNUDGE and it was called as an ensemble model as depicted in Taslim and Lin (2014) and was fused together by means of incorporating a SVD method for the estimation of robust parameters in this technique, thereby providing the term FHM for the proposed model. A versatile weighting scheme was also added to this fusion model. To enhance the flexibility of this fusion model based on the location and scale values, the differentiated observations are captured and classification of some of the normal components is allowed. Among the various classes, the overall model is selected, and the inference is provided, as it depends on the underlying distribution of the data.

### Fusion Model of Finite Mixtures

The multiple underlying observations are considered, and a fusion approach is developed, as it integrates the merits from various models. The various classes of mixture models are collectively utilized. To fit the normalized data, the design of each class of model is utilized and projected as differences between various experimental situations. For the normalized data point *z*, *f*(*z*) is assumed as the unknown density function and is expressed as follows:


(1) $$f\left(z;\Psi \right)=\left(1-\pi \right)f_{0}\left(z;\Psi_{0}\right)+\pi f_{1}\left(z;\Psi_{1}\right)$$

For the mixture, the underlying model parameters are expressed as Ψ, Ψ_0_, and Ψ_1_, respectively. To capture the overdispersion elements, the designation of *f*_1_ is done, and to identify the more centrally situated ones, *f*_0_ is utilized. To trace the differential observations also, *f*_0_ is utilized effectively. The modeling of *f*_1_ and *f*_0_ is expressed as follows (Taslim and Lin, 2014):


(2) $$f_{1}\left(z;\Psi_{1}\right)=\left\{ \begin{array}{c} V_{\left(i,j\right)}\left(z\right),\begin{array}{cc} & \begin{array}{ccc} & & for \end{array}e\;NUDGE \end{array}\\ \rho E_{1}\left(-z\times Ind\left\{z<-\xi_{1}\right\};\beta_{1}\right)+\left(1-\rho \right)\\ \times E_{2}\left(z\times Ind\left\{z>\xi_{2}\right\};\beta_{2}\right),\begin{array}{cc} for & GNG \end{array} \end{array}\right.$$

(3) $$f_{0}\left(z;\Psi_{0}\right)=\left\{ \begin{array}{c} N\left(z;\mu ,\sigma^{2}\right)\sum_{q=1}^{Q}\gamma_{q}\times N\left(z;\mu_{q},\sigma_{q}^{2}\right),\begin{array}{cc} for & NUDGE \end{array}\\ \sum_{q=1}^{Q}\gamma_{q}=1,\begin{array}{cc} & \begin{array}{cccc} & & for & eNUDGE+GNG \end{array} \end{array} \end{array}\right.$$

With the help of a uniform distribution or with the help of a mixture of two exponential distributions, the capturing of overdispersion in the data is done (Taslim and Lin, 2014). As a section of the model parameters, *i* and *j* are considered as parameters of the uniform distributions [i.e., (*i*, *j* ∈ Ψ_1_)]. The model parameters also include the blending measure of the exponential distributions and the scale specifications, i.e., ρ, β_1_, β_2_ ∈ Ψ_1_.

Let *ξ*_1_, *ξ*_2_ which are greater than zero be considered and known as the location parameters. The estimator of *ξ*_1_ and *ξ*_2_ is utilized by ${\hat{\xi }}_{1}=\left|\max\left(z<0\right)\right|$ and ${\hat{\xi }}_{2}=\left|\min\left(z>0\right)\right|$. With the help of normal distributions (single or mixture), the representation of more centrally situated data is done. As a section of model parameters, the location and scale parameters are involved here (i.e., $\mu ,\sigma^{2},\mu_{q},\sigma_{q}^{2}\in \Psi_{0}$). As a section of model parameters, the number of components in the mixture *Q* and the mixing proportions γ_*q*_ are also involved, i.e., γ_*q*_, *Q* ∈ Ψ_0_. Therefore, Ψ = {π}∪Ψ_0_∪Ψ_1_. If the condition in {} is proved, then the Indicator function *Ind*{.} is equal to one or else it is zero. In the normal mixture, some terms along with it will be identified as “differential,” as any distribution can be identified by a specific combination of normal distributions.

### Singular Value Decomposition-Based Estimation of Robust Parameters

For the model parameters, to obtain a good estimation in the fusion model, a weighted likelihood function is utilized as follows:


(4) $$ml\left(\Psi \right)=\sum_{k=1}^{m}wei_{k}\log f\left(z_{k};\Psi \right)$$

where for*z*_*k*_, *k* = 1, 2, …, *m* are the normalized data and the prespecified weights mentioned as 0 ≤ *wei*_*k*_ ≤ 1.

The SVD (Martinsson, 2012) is then implemented to obtain the Eigen value observations. From the Eigen value observations, the contributions are downgraded by means of using small intensities, and so, the weighted likelihood is utilized. With the similar log-ratio, the data points are distinguished, but at the same time, various magnitudes are present for their respective intensities. The average low intensities be considered as *v*, and therefore, the lower half Herbert’s neglect function is expressed as follows:


(5) $$wei\left(v\right)=\left\{ \begin{array}{c} 1,if\begin{array}{cc} v>-g & \end{array}\\ \frac{g}{\left|v\right|},if\begin{array}{cc} v\leq -g & \end{array} \end{array}\right.$$

where g = 1.5 and is utilized to down weight those factors with less intensities. Under this fusion approach, the fitting of each model class is utilized by the standard Expectation Maximization (EM) algorithm. When a maximum number of iterations *T*are reached or when ||Ψ_(*t* + 1)_−Ψ_(*t*)_|| < ε, then the stop criterion of EM algorithm is achieved. The values used in this experiment are chosen after several trial-and-error measures based on performance, and finally, ε = 10^−8^ and *T* = 1,000 is used in our fusion approach.

### Model Selection and Model-Based Identification of Features

In the proposed model, the total number of normal components *Q* must be determined. The models are examined with *Q* = 1, 2, … and then *Q* is chosen so that Bayesian Information Criteria (BIC) is maximized (Lin et al., 2015). Within each class, the best model is identified, and then, the overall best model is selected by Akaike Information Criteria (AIC) (Tharmaratnam and Claeskens, 2013). By using this kind of a balanced model selection, too complex models or too simple models can be avoided easily. A two-step technique is utilized by the selection of best model to identify every observation as a differential category or not. A normal component $Norm\left(\mu_{q},\sigma_{q}^{2}\right)$ is identified as a differential one in the initial step if the capturing of observations is done as outliers in the overall distribution as follows:


(6) $$\left|\mu_{q}\right|+2\times \sigma_{q}>1.5\times IDR$$

where IDR represents the interquartile range for the dataset.

The non-differential category is the one in which the labeling of the normal component is not done as a differential one. For every observation, once the labeling of every normal component is done, then the false discovery rate (FDR) is computed as follows:


(7) $$FDR\left(z_{k}\right)=\frac{f_{norm}\left(z_{k},{\overset{⌢}{\Psi }}_{0}\right)}{f\left(z_{k};\overset{⌢}{\Psi }\right)},\forall_{k}\in m,$$

where *f*_*norm*_ comprises of normal components that are termed as non-differential. For any threshold point *y*_0_, the observation *z*_*k*_ is identified with weight *wei*_*k*_ to be a distinctive element if *FDR*(*z*_*k*_)/*wei*_*k*_ ≤ *y*_0_. Thus, the redundant components are completely eliminated and only the essential fusion hybrid modeled features are retained in the model.

Table 1 exhibits the analysis of statistical parameters for FHM models in various datasets. The statistical parameters are vital to extract the non-linear nature of the underlying physiological events. Here, six parameters, namely, mean, variance, skewness, kurtosis, sample entropy, and permutation entropy are calculated for the epilepsy dataset (Andrzejak et al., 2001) and schizophrenia datasets, namely (Olejarczyk and Jernajczyk, 2017), under FHM models. When utilizing the FHM model, the mean parameters are found with common ground values. In the variance and permutation entropy as well, there is not much variation among the models across the datasets. The skewness and kurtosis indicate the presence of non-linearity and non-Gaussian conditions among the datasets. Sample entropy is distinguished itself as a parameter of wide variation among the datasets and models. This finely indicates the presence of a non-peaked Gaussian density due to the feature extraction models and therefore feature selection is absolutely necessary.

**TABLE 1:** Analysis of statistical parameters for Fusion Hybrid Model (FHM) in various biosignal datasets.

Parameters	A-E	B-E	C-E	D-E	AB-E	CD-E	Schizophrenia
Mean	0.38376	0.81425	0.196404	0.689723	1.262721	0.082903	0.49361
Variance	0.107887	0.000832	0.038601	0.01086	0.003261	0.002902	0.009318
Skewness	2.641229	0.015824	0.936748	–0.08714	–2.08109	7.461258	0.160928
Kurtosis	13.30222	–0.07862	–0.75852	0.159329	4.590378	75.3816	–0.92298
Sample entropy	11.9076	6.2048	1.9800	6.9953	7.4203	9.8152	6.4148
Permutation entropy	1.5993	1.522	1.7095	1.7344	1.7407	1.4749	1.0926

## Proposed Feature Selection Technique Using Hybrid Differential Particle Artificial Bee

**FIGURE 2:**
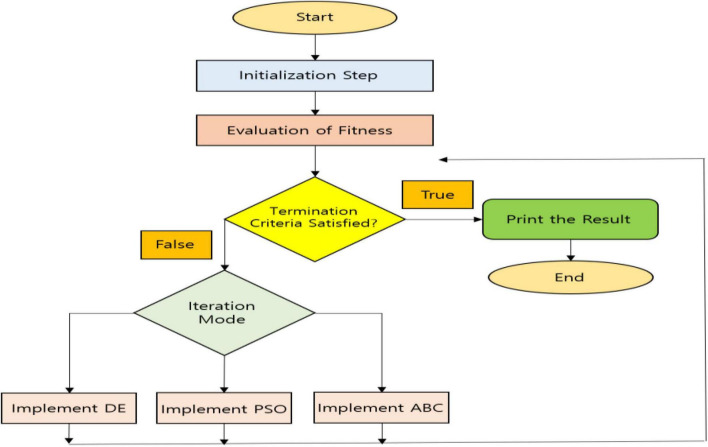
Pictorial representation of Hybrid Differential Particle Artificial Bee (HDPAB).

Once the features are extracted using the FHM model, then, the features have to be selected before feeding inside classification. For solving real-world optimization issues, the hybrid algorithms are of great use, as a better or high-quality solution can be obtained. A flourishing concept in the field of artificial intelligence (AI) is swarm intelligence, and it is the collective behavior of natural or artificial self-organized systems (Prabhakar et al., 2019). Some of the commonly used swarm intelligence techniques with its applications to various domains are reviewed in Yang (2014). In this study, the concept of a hybrid swarm intelligence technique is proposed as an efficient feature selection technique. To solve various issues in different fields, many metaheuristic algorithms are utilized such as Central Force Optimization, Lightning Attachment Procedure Optimization, Genetic Bee Colony Optimization, etc. (Prabhakar and Lee, 2020a). To trace the global optimum point by means of achieving both exploration and exploitation qualities, these algorithms are used. Selecting the control parameters and adjusting or fine tuning it is a very important stage in these procedures (Prabhakar and Lee, 2020b). To get a solution with high quality, sometimes exploitation should be more than exploration or *vice versa* depending on the problem characteristics. Various properties are utilized by every optimization algorithm so that the appropriate goals are achieved. For some specific problems, some optimization algorithms perform better and some specific problems as well as some optimization algorithms perform worse. Therefore, many techniques are combined to address these difficulties in literature and are known as hybrid models. To have a steady balance between the exploitation and exploration qualities, control parameters utilized by the optimization algorithms should be perfect. To solve the optimization problems, solution quality is also very important. Sometimes, the algorithm may be very versatile and robust, but it can have a very low solution quality, and sometimes, the algorithm may be less versatile and robust, but it can have a better solution quality. Therefore, to achieve both the control parameters and high-quality solution, hybrid algorithms are proposed. The proposed algorithm is called HDPAB algorithm, and it utilizes the hybridization of differential evolution (DE) (Mohamed, 2015), particle swarm optimization (PSO) (Altamirano and Riff, 2012), and Artificial Bee Colony Optimization (ABC) (Karaboga and Basturk, 2008) to select the most important features, and the pictorial representation is expressed in Figure 2.

### Proposed Hybrid Differential Particle Artificial Bee Algorithm

A powerful algorithm called HDPAB is generated in the context of both robustness and high-quality solution. To select the control parameters of algorithms, random choosing of parameters from the required ranges is done. Without altering the characteristics, the primary operators of the designated algorithms are utilized by HDPAB. To select the candidate solutions, the implementation of operators of the merged techniques in a repeated mode is performed. Therefore, to select a particular algorithm, the suitable operators are implemented, and a number of solutions are found, and to the next algorithm, a new population is incorporated.

**Step 1:** The candidate populations of solutions *A*_*i*_ is initialized, where *i* = {1, 2, 3, …, *NP*}. The size of the population is denoted by *NP*.

**Step 2:** With the following mathematical expressions, the crossover, mutation, and selection operators of DE algorithms are implemented as follows:


(8) $$Y_{i}=A_{x}+G\left(A_{z}-A_{w}\right)$$

(9) $$V_{ij}=\left\{ \begin{array}{c} Y_{ij},\begin{array}{cc} if & n_{j}\leq CR \end{array}\\ A_{ij},\begin{array}{cc} if & otherwise \end{array} \end{array}\right.$$

(10) $$A_{i}=\left\{ \begin{array}{c} V_{i},\begin{array}{cc} if & f\left(V_{i}\right)<f\left(A_{i}\right) \end{array}\\ A_{i},\begin{array}{cc} & \begin{array}{cc} & \begin{array}{cc} & otherwise \end{array} \end{array} \end{array} \end{array}\right.$$

For every member in the candidate population, using Equation (8), the calculation of the mutant vector *Y*_*i*_ is done. In this population, the distinct members are considered as *A*_*x*_, *A*_*z*_, and *A*_*w*_. By using Equation (9), to generate *V*_*ij*_, the crossover operator *Y*_*ij*_ is crossed with *A*_*ij*_, where the *j^th^* elements of the *i^th^* solution vector *A*_*i*_ are denoted by *Y*_*ij*_ and *A*_*ij*_ respectively. For every *j^th^* element of *Y*_*i*_, a uniformly distributed number is represented as *n*_*j*_. For the DE, the main control parameters are *G* and *CR*; for the sake of mutation and crossover operation, they are used. The determination of the novel candidates for *A*_*i*_ is done in the selection process depending on the fitness value of *V*_*i*_ and *A*_*i*_ by using Equation (10), where it is assessed either using the vector *V*_*i*_ or its preceding solution.

**Step 3:** Utilizing the following expression, the PSO algorithm operators are applied as follows:


(11) $$Velo_{i}=qVelo_{i}+w_{1}\left(P_{best,i}-A_{i}\right)+w_{2}\left(global_{best}-A_{i}\right)$$

(12) $$A_{i}=A_{i}+Velo_{i}$$

By utilizing Equation (11) to the *Velo*_*i*_, the main control parameters are implemented as *q*, *w*_1_, and *w*_2_. Using Equation (12), the positions are updated using these particles. The best-known position is extended, and that point is known as *global*_*best*_ and the best position extended by the *i^th^* particle in the swarm is expressed as *P*_*best*, *i*_

**Step 4:** The operator of ABC algorithm is performed by means of implementing control parameters in order to update the respective candidate positions in search space. In a situation in which the further movement of the position cannot be guaranteed, then Equation (13) is utilized so that a new food source utilized by the scouts is used to replace the food source, which had been abandoned in the nectar by bees. Within a specific range of cycles, to assess the abandonment of food sources, the control parameter utilized is called “limit” in ABC algorithm.


(13) $$a_{i}^{j}=a_{\min}^{j}+rand\left(0,1\right)\left(a_{\max}^{j}-a_{\min}^{j}\right)$$

**Step 5:** Unless a chosen stopping criterion is satisfied, the steps (9), (10), and (11) are repeated. Unless the termination criterion is satisfied, the performance of the algorithm in a loop is done. At the end of every iteration, the best solution is preserved, and therefore, elitism is incorporated in HDPAB, thereby selection of the most important features suitable for classification is obtained.

## Development of Classification Models

The selected features have to be fed inside the classification models for the sake of classification of the healthy vs. the diseased ones. In this section, initially, the ZIPMRM for biosignal processing is developed, then suitable deep learning models are also developed, and finally, the results are analyzed with the standard machine learning techniques as well. A mixture model is a simple probabilistic model, which indicates the occupancy of sub-populations within a comprehensive population (Mclachlan, 2005). For the distribution of the mixture components, the familiar prospects are generally binomial distribution, multinomial distribution, log-normal distribution, multivariate t-distribution, negative binomial distribution, Poisson distribution, exponential distribution, etc. (Ueda et al., 2000). Various models are available in literature such as Gaussian Mixture Models (GMM) (Constantinopoulos and Likas, 2007), Hidden Markov Model (HMM) (Krogh et al., 1994), Categorical Mixture Model (CMM) (Kanzawa, 2017), Finite Mixture Model (FMM) (McLachlan and Peel, 2000), Multivariate Gaussian Mixture Model (MGMM), etc. (Scrucca et al., 2017), and they have been generally used for biomedical signal processing applications unlike the PMRM. The application of mixture models has been utilized in analyzing financial models, handwriting recognition, predictive maintenance, fuzzy image segmentation, house price evaluation, point set registration, etc. (Elguebaly and Bouguila, 2011). For the estimation of parameters in the mixture models, the commonly used techniques are EM algorithm, Markov Chain Monte Carlo, Graphical Methods, moment matching, and spectral methods (Meignen and Meignen, 2006). PMRM has been widely used in the literature for various purposes such as analysis of count data (Wang et al., 2007), biometrics (Wang et al., 1996), detection of maternity duration of hospital stays (Wang et al., 2002), financial data modeling and classification (Faria and Goncalves, 2013), defaulters behavior approach analysis (Karlis and Rahmouni, 2007), insurance ratemaking (Bermudez and Karlis, 2012), and heart disease prediction (Mufudza and Erol, 2016), but no literature has been available with respect to utilizing it for the classification of biomedical signals and so an attempt was made in this study successfully.

### Proposed Zero Inflated Poisson Mixture Regression Models

In this section, a general introduction to the mixture regression model, followed by the mixture of regression classes, and implementation of PMRM followed by the assessment of the proposed ZIPMRM for classification of the features are explained.

#### Mixture Regression Model

In the data acquisition procedure, there might be omission of certain vital covariates, and therefore, the occurrence of unobserved heterogeneity is high in regression. In such cases, the main features are not taken into consideration and thereby it leads to the estimation of biased parameter values. The homogeneous observations are grouped into certain categories or clusters so that the various heterogeneity issues can be solved by the mixture regression models (Wang et al., 1996). The uniform observations can be standard, fixed, or having saturated variables. The calculation of posterior probabilities has a profound influence on the mixture regression models for the specific variables. Depending on the discriminant analysis rule, the grouping of the standard variable models is done followed by the classification. For the mixture distribution, the joint distribution is mentioned and the response to the conditional distribution is analyzed. By utilizing mixture regression models for the distributions of parameter, the normality is relaxed, as it utilizes a generalized linear model (Wang et al., 2002). A mixture of *q* components is assumed in a finite mixture regression composition, where a specific parametric distribution is traced by every component in the model. For every observation, a weight probability is assured by all the components so that the weighted summation value over the *q* components is expressed by the mixture distribution. The classification can be improved in most segmented cases with the help of this mixture model. To the clustering model, a very high heterogeneous composition can be implemented if the regression parameters are assumed to be relaxed for the generalized linear model.

#### Mixture Regression Classes

When analyzing the mixture regression models, two classes, namely, (a) standard variable mixture regression models and (b) concomitant values mixture regression models can be considered. A generalized linear model with similar error and link function is used to describe the component here, and it is done with various linear operators. For a generalized linear model, a standard mixture regression is expressed as follows:


(14) $$f\left(u|v,\Phi \right)=\sum_{q}\pi_{q}f_{q}\left(u|v;\beta_{0q},\beta_{q}\right)$$

where *u* describes the response variable with an exponential distribution, which is dependent on component *q*. For the response variable, the conditional expectation is expressed as follows:


(15) $$E\left(u|v\right)=h^{-1}\left(\beta_{0q}+v^{\prime }\beta_{q}\right)$$

where *h*(.) expresses the link function. Here, Φ = {β_0_, β, π}, β_0_ = (β_0*q*_), β = (β_*q*_). The commonly used probability distributions are Poisson, geometric, normal, binomial, and negative binomial (Faria and Goncalves, 2013). In the analysis of data, the nature of the counts should be analyzed, and so, the Poisson distribution is considered here. For every component involved, the assumption is that for the same parametric family, component-specific densities are considered, that is, *f*_*q*_ = *f* for simple notation representation. For all the components, the link function is similar and expressed as *h*_*q*_ = *h*. There is no prior knowledge about the distribution of family differences in this cluster-wise regression model.

For the prior class probabilities, the concomitant variable model forms as a popular extension, so that based on the set of explanatory variables, the weight π_*q*_ can be fully dependent on it. The random effects do not have any influence on the data distributions and on the parameter estimates, and therefore, the substantial changes are not required. Depending on a regression model based on Equation (14), a concomitant variable mixture regression model is framed, which utilizes posterior probability parameterization so that it is fit into two distinct sets *v* = (*v*_1_, *v*_2_) and the range of concomitant variables can be conquered easily.

The initial set influences *u* along with the latter set influences the latent group specifier *w* variable as follows:


(16) $$f\left(w|v;\pi \right)=\pi_{q}\pi_{q|v}$$

For the response variable, the marginal distribution is expressed as follows:


(17) $$f\left(u|v_{1},v_{2};\Phi \right)=\sum_{q}\pi_{q|v_{2}}f_{q}\left(u|v_{1};\beta_{0q,}\beta_{q}\right)$$

Here, Φ = {β_0_, β, γ_0_, γ}, β_0_ = (β_0*q*_), β = (β_*q*_), γ_0_ = (γ_0*q*_) and γ=(γ)q.

The component indicator functions are used to derive γ_*q*_, which are expressed as follows:


(18) $$\pi_{q|v}=\frac{\exp\left(\gamma_{0q}+v^{\prime }\gamma_{q}\right)}{\Sigma_{g}\exp\left(\gamma_{0g}+v^{\prime }\gamma_{g}\right)}$$

Its identification is usually considered as zero. With the help of model intercept, the aliasing of the intercept parameters β_0_ is done, which results in avoidance of the huge standard errors.

#### Poisson Mixture Regression Model Implementation

In this model, the poison density function of a model, which has a distribution with response *U* and covariate vector *v*, is expressed as


(19) $$f\left(u,\lambda \right)=\frac{e^{-\lambda }\lambda^{u}}{u!}Ind_{B}\left(u\right)$$

In Equation (19), *E*(*u*) = λ, the link function *h*(λ) = *log*⁡(λ) = β*^T^v*, *B* = {0, 1, 2, …}, represents the non-negative integer set and *Ind*_*B*_(*u*) represents the indicator function. If u′′belongs to the set *B*, the value becomes one, otherwise its value is zero. The variance *var*(*U*) = *E*(*U*) = λ, and dispersion issues are prevalent if *var*(*U*) > *E*(*U*) for overdispersion and *Var*(*U*) < *E*(*U*) for underdispersion. Underdispersion is less common than overdispersion (Karlis and Rahmouni, 2007). When analyzing the Poisson distribution, if the count data are relatively overdispersed, then it leads to misleading results. Therefore, extradispersion problems have to be dealt more carefully. In a population in which there is too much unobserved heterogeneity, the appropriate finite mixture model is utilized, which has two more sub collective groups and are highly mixed in different proportions.

When covariate information is present, the fitting of the extradispersed data can be done in PMRM provided the objectives are assumed to be obtained from a finite mixture. Sometimes, the distribution can vary in different intercepts and the explanatory variable is presumed to have a heterogeneous nature. The clustering of the count outcomes is considered and then the regression model is incorporated with random effects so that in between the observations, an inherent correlation can be observed. Some of the common Poisson Mixture Models (PMM) are Zero Inflated Poisson (ZIP), negative binomial, and Zero Inflated Negative binomial (ZINB) models, respectively (Mufudza and Erol, 2016). To manage heterogeneity and the additional zeros in the data, the ZIP model is usually utilized. Depending on Poisson distribution, a finite mixture model can be expressed as follows:


(20) $$f_{k}\left(u_{i},\theta_{ik}\right)=\frac{e^{-\lambda_{ik}}{\left(\lambda_{ik}\right)}^{u_{i}}}{u_{i}!}Ind_{B}\left(u_{i}\right)$$

In Equation (20), $\log\lambda_{ik}=\beta_{k}^{T}v_{i},i=1:m,k=1:l$ and for the *l* component mixture,


(21) $$E\left(u_{i}\right)=\sum_{i=1}^{l}\pi_{k}\lambda_{ik}$$

(22) $$Var\left(U_{i}\right)=E\left(var\left(U_{i}|W_{i}\right)\right)+var\left(U_{i}|W_{i}\right)=E\left(U_{i}\right)+y_{ik}$$

where $\lambda_{ik}=\exp\left(\beta_{k}^{T}v_{i}\right)$ represents the mean of the *i^th^* responsive situation to its respective membership in the *k^th^* constituent of the mixture, $y_{ik}=\Sigma \pi_{k}\lambda_{i_{k}}^{2}-{\left(\Sigma \pi_{k}\lambda_{i_{k}}\right)}^{2}$ and *W*_*k*_ represents the component specifier of zeros and ones. Here, *W*_*ik*_ = (*W*_*k*_)*i* belongs to one if it comes from component *i* and zero otherwise. *y_i_k__* = 0, if λ_1*i*_ = λ_2*i*_ = …λ_*li*_, specifying that more than a homogeneous model, the mixture model performs much better. For PMRM, the heterogeneity is varied across individuals is such a manner that it has a discrete mixture distribution.

#### Assessment of a Zero Inflated Poisson Mixture Regression Model

Depending on the total number of counts being generated in the model, the data with many zeros can be served with the help of ZIPMRM. To manage both the zeros and heterogeneity, ZIPMRM acts as a very special mixture regression case. It comprises of a normal count distribution, which can be either a negative binomial or Poisson along with binary distribution, which can be degenerated at zero. In the analysis, the zeros may or may not be included with the non-zeros. This mechanism is considered as a dual data-generating mechanism, where zeros are generated from one side and a full range of counts is generated from the other side. Here, the consideration of ZIP model is done as there were structured zeros inside the data, which is indicated as true zeros in terms of the counting process. The assumption in the ZIP model is as follows:


(23) $$u_{i}=\left\{ \begin{array}{c} 0,if\;\begin{array}{cc} feature_{i}=0 & \end{array}\\ Poisson\left(\lambda_{i},\beta ,\gamma \right),\begin{array}{cc} if & feature_{i}=1 \end{array} \end{array}\right.$$

where *feature*_*i*_ indicates whether the constituent *i* has the disease or not (i.e., epileptic or not, schizophrenic or not).

### Deep Learning Model Using Bi-Long Short-Term Memory

**FIGURE 3:**
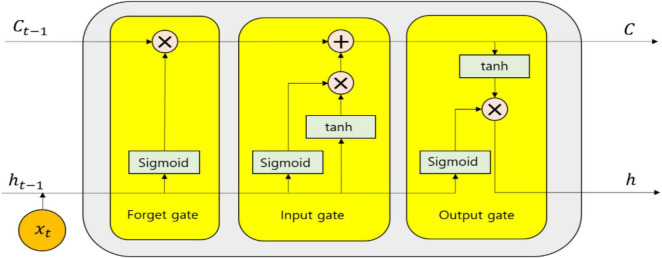
Simple representation of an Long Short-Term Memory (LSTM) unit.

By means of considering the selected features, the classification is also done by means of utilizing a deep learning model with the help of BiLSTM where a bidirectional recurrent network is constructed by means of utilizing LSTM units. The past and future information can be captured easily so that the features can be classified by means of utilizing non-linear functions (Chen et al., 2017). BiLSTM comprises of both forward LSTM and backward LSTM. The information, which is not useful for classification, is omitted completely by LSTM and only the valuable information is passed to the future time point. Every LSTM unit comprises of an input gate, an output gate, and a forget gate as projected in Figure 3.

In the LSTM unit, the transmission of information is dependent on the forget gate. To assess whether the information is useful or not for detection or classification of the disease, it receives the previous hidden state *h*_*t*−1_ and the current information *x*_*t*_ continuously. To obtain the current cell state *C*, the effective utilization of the previous cell state *C*_*t*−1_, previous hidden state *h*_*t*−1_ along with the current information *x*_*t*_ is done by the input gate. The output gate is utilized to provide the probability of two cases (epileptic or not, schizophrenia or not) by means of utilizing current information or previous information. The calculation of each gate at time *t* for every LSTM unit is expressed as follows:


(24) $$i_{t}=\delta_{g}\left(W_{i}.\left[h_{t-1},x_{t}\right]+b_{i}\right)$$

(25) $$o_{t}=\delta_{g}\left(W_{o}.\left[h_{t-1},x_{t}\right]+b_{o}\right)$$

(26) $$f_{t}=\delta_{g}\left(W_{f}.\left[h_{t-1},x_{t}\right]+b_{f}\right)$$

(27) $$C_{t}=f_{t}C_{t-1}+i_{t}\delta_{c}\left[W_{c}.\left[h_{t-1},x_{t}\right]+b_{c}\right]$$

(28) $$h_{t}=o_{t}\delta_{c}\left(C_{t}\right)$$

where the sigmoid function is represented as δ_*g*_, the cell states are expressed by *C*, hyperbolic tangent function is expressed by δ_*c*_, and the hidden states is denoted by *h*. The formulation of the forget gate, output gate, and input gate is done in terms of *f*, *o*, and *i*, respectively. The weight of each gate is represented as *W* and the bias value is specified by *b*. By utilizing the current signal value *x*_*t*_ along with the previous hidden state *h*_*t*−1_, the computation of the input of each function is done.

**FIGURE 4:**
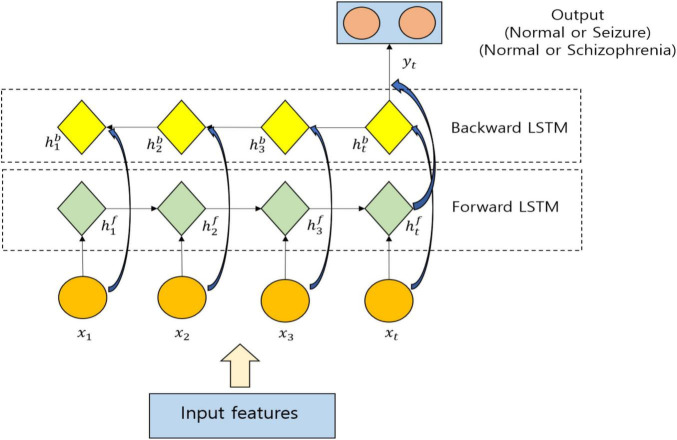
Utilization of Bi-Long Short-Term Memory (LSTM) for classification.

The EEG-based epileptic/schizophrenia classification by means of utilizing Bi-LSTM is illustrated in Figure 4, in which the input is nothing but the selected features. *x*_*t*_ specifies the value at *t*. The hidden state of the forward LSTM is specified by $h_{t}^{f}$ and the hidden state of the backward LSTM is specified by $h_{t}^{b}$. The features are processed by the forward LSTM from left to right and the hidden layers are utilized to pass the information. Based on the current input *x*_*t*_ and the previous hidden state $h_{t-1}^{f}$, the computation of the current hidden state $h_{t}^{f}$ is done. The future information is passed to the history unit by the backward LSTM, and based on the input *x*_*t*_ along with the current hidden state $h_{t}^{b}$, the calculation of the previous hidden state $h_{t-1}^{b}$ is done. The weights of Bi-LSTM are formulated as *W* = {*W*_*f*_, *W*_*i*_, *W*_*o*_, *W*_*c*_}. The cross entropy is utilized to establish the loss function of the Bi-LSTM. For minimizing the loss function *L*, the training of the model is done to compute the weights *W* and bias *b*. To update the parameters *W* and *b*, the utilization of gradient descent algorithm is done where it is formulated as follows:


(29) $$W=W-\eta \frac{\partial L}{\partial W}$$

(30) $$b=b-\eta \frac{\partial L}{\partial b}$$

where the learning rate is indicated by η. The utilization of the backward propagation algorithm is done so that the parameters *W* and *b* at every layer are updated. The final output of the Bi-LSTM is followed by means of using a Fully Connected (FC) Layer, wherein the activation function utilized is softmax and this is done so that the mapping of the inputs is done successfully into the probability values. The softmax function is expressed as follows:


(31) $$y_{j}^{\left(i\right)}=\frac{e^{z_{j}^{\left(i\right)}}}{\sum_{j=1}^{C}e^{z_{j}^{\left(i\right)}}}$$

## Results and Discussion

The FHM is utilized for the sake of extracting the features in terms of parameter components from the EEG signals. The features are selected with the help of a hybrid swarm algorithm and later classified with the developed classification models. The proposed model has been validated on EEG datasets and the following results have been obtained in terms of classification accuracy. The EEG datasets considered were Bonn Epileptic dataset, which has five subsets such as set A, set B, set C, set D, and set E, respectively (Andrzejak et al., 2001) along with a Schizophrenia dataset (Olejarczyk and Jernajczyk, 2017). All the in-depth details of the datasets are provided in the respective reference links. As far as the epileptic dataset is considered, Set A and B are obtained from five healthy volunteers; Set C, D, and E are obtained from five epileptic patients. The recorded period of Set A and B belongs to normal category and the recorded period of Set C and D represents the inter-ictal state (seizure free intervals) and Set E represents the ictal state (seizure activity). Six classification problems were studied for the epileptic dataset such as A-E, B-E, C-E, D-E, AB-E, CD-E, and for schizophrenia dataset, the classification study is between healthy vs. schizophrenia patients. The signal datasets were initially preprocessed using independent component analysis (ICA) before implementing the proposed methodology. A total of [4,097 × 100] data are present in the epileptic dataset, and initially when the features are extracted by FHM model, it is reduced to [2,500 × 50], and when the essential features are selected by HDPAB, it is reduced to [1,500 × 25] and it is fed to classification. Similarly, in schizophrenia dataset, 225,000 samples are present, which is split into collections of 5,000 sample segment, wherein each channel represents the data with a matrix of [5,000 × 45] per patient and for all the 19 channels, it is represented as [5,000 × 45 × 19]. When the features are extracted by FHM model for a single channel, it is reduced to [2,500 × 35], and when the essential features are selected by HDPAB, it is reduced to [1,500 × 25] and it is fed into classification. The classification of dataset is in such a way that 80% is training data, 10% is validation data, and 10% is test data. A 10-fold cross-validation technique is utilized for the ZIPMRM, deep learning, and machine learning models. Randomly, the dataset which is to be classified is split into 10 different parts. As usual, nine parts of the data were utilized for training and validation purposes and the rest one part was utilized for testing purposes. This process was repeated for about 10 times so that under every circumstance, different parts of training, testing, and validation data can be used effectively. Other hyperparameter settings for the deep learning are as follows: The optimizer used is Adam, learning rate is set at 0.01, the batch size is set at 40, and the number of hidden units is set as 80. The L2 regularization rate is set as 10^−4^. The other classifiers utilized for comparison along with the deep learning models are K-Nearest Neighbor (KNN), Naïve Bayesian Classifier (NBC), Adaboost, Linear Discriminant Analysis (LDA), SVM-RBF, Quadratic Discriminant Analysis (QDA), HMM, and GMM.

Table 2 expresses the performance analysis of FHM and HDPAB for different datasets in terms of accuracy. When the KNN classifier is considered, for the all datasets, a minimum classification accuracy of 76.20% was obtained for AB-E dataset and a high classification accuracy of 83.98% was obtained for schizophrenia dataset. Similarly, when NBC is considered, a low classification accuracy of 77.21% was obtained for A-E dataset and a high classification accuracy of 85.54% was obtained for schizophrenia dataset. The other techniques such as Adaboost, LDA, and QDA are performed in an average manner producing accuracies in the range of seventies and eighties. SVM classifier performed well by producing a high classification accuracy of 95.67% for schizophrenia dataset and a low classification accuracy of 91.08% for AB-E dataset. Similarly, HMM and GMM also performed in an above average manner, as the classification accuracies are found in the range of eighties. The ZIPMRM too performed well by producing a high classification accuracy of 95.31% in the A-E dataset and a low classification accuracy of 91.82% in the C-E dataset. Finally, when classified with LSTM and Bi-LSTM, the performance is exemplary, as a very high classification accuracy is obtained easily, with LSTM reporting a higher accuracy of 98.61% for the A-E dataset and a lower accuracy of 94.92% in the D-E dataset. Similarly, Bi-LSTM reports the highest classification accuracy of 98.79% in the case of A-E dataset and a comparatively low accuracy of 97.14% in the case of AB-E dataset, thus concluding that it performs supremely well when compared with the other conventional classifiers.

**TABLE 2:** Performance analysis of Fusion Hybrid Model (FHM) and Hybrid Differential Particle Artificial Bee (HDPAB) for different datasets in terms of accuracy.

Classifier	A-E	B-E	C-E	D-E	AB-E	CD-E	Schizophrenia
KNN	80.11	76.67	77.34	80.49	76.20	80.88	83.98
NBC	77.21	79.82	77.92	84.33	77.35	84.22	85.54
Adaboost	87.42	87.82	87.58	87.10	89.37	85.36	88.47
LDA	81.67	77.53	80.01	86.71	83.78	77.39	77.14
SVM	96.88	93.89	95.97	92.93	91.08	94.6	95.67
QDA	81.47	85.81	83.34	84.05	82.02	84.85	88.59
HMM	92.31	91.92	88.11	89.18	89.37	85.11	86.11
GMM	82.31	79.12	81.29	82.38	81.83	78.59	79.71
ZIPMRM	95.31	92.58	91.82	92.47	93.59	93.48	94.83
LSTM	98.61	96.31	96.61	94.92	95.71	96.82	97.49
Bi-LSTM	98.79	97.71	98.66	98.75	97.14	96.49	98.35

### Comparison of Our Results With the Previous Studies

The results with the proposed flow of methodology are compared with the existing studies and are shown in Table 3 for the Bonn epileptic dataset and schizophrenia dataset. Few studies are available in the literature regarding schizophrenia classification because the research is still under progress, while as far as the epilepsy classification is concerned, the field is more established and so some of the prominent studies in recent years are compared with our results.

**TABLE 3:** Comparison study with the works reported on the similar datasets used for both epilepsy classification and schizophrenia classification.

Classification issue dealt	References	Technique utilized	Classification accuracy (%)
A vs. E	Samiee et al., 2015	Discrete Short Time Fourier transform with Multilayer Perceptron	99.80
	Bhardwaj et al., 2016	EMD combined with genetic programming	98.64
	Diykh et al., 2017	Establishing a weighted complex network with SVM classification	100
	Sharma et al., 2018	Orthogonal wavelet implementation with SVM	100
	Proposed method	FHM + HDPAB+ ZIPMRM	95.31
		FHM + HDPAB+ LSTM	98.61
		FHM + HDPAB +BiLSTM	98.79
B vs. E	Diykh et al., 2017	Establishing a weighted complex network with SVM classification	99.76
	Sharma et al., 2017	Analytic Time Frequency Flexible Wavelet Transform with SVM	82.88
	Nicolaou and Georgiou, 2012	Permutation entropy with SVM	93.55
	Proposed method	FHM + HDPAB+ ZIPMRM	92.58
		FHM + HDPAB+ LSTM	96.31
		FHM + HDPAB +BiLSTM	97.71
C vs. E	Samiee et al., 2015	Discrete Short Time Fourier transform with Multilayer Perceptron	98.50
	Diykh et al., 2017	Establishing a weighted complex network with SVM classification	96.00
	Sharma et al., 2017	Analytic Time Frequency Flexible Wavelet Transform with SVM	99.00
	Nicolaou and Georgiou, 2012	Permutation Entropy with SVM	88.00
	Proposed method	FHM + HDPAB+ ZIPMRM	91.82
		FHM + HDPAB+ LSTM	96.61
		FHM + HDPAB +BiLSTM	98.66
D vs. E	Samiee et al., 2015	Discrete Short Time Fourier transform with Multilayer Perceptron	94.90
	Diykh et al., 2017	Establishing a weighted complex network with SVM classification	93.70
	Nicolaou and Georgiou, 2012	Permutation Entropy with SVM	79.94
	Riaz et al., 2016	EMD and SVM	93.00
	Proposed method	FHM + HDPAB+ ZIPMRM	92.47
		FHM + HDPAB+ LSTM	94.92
		FHM + HDPAB +BiLSTM	98.75
AB-E	Diykh et al., 2017	Establishing a weighted complex network with SVM classification	96.40
	Raghu et al., 2019	Analysis of Matrix Determinants with Multilayer Perceptron	97.10
	Proposed method	FHM + HDPAB+ ZIPMRM	93.59
		FHM + HDPAB+ LSTM	95.71
		FHM + HDPAB +BiLSTM	97.14
CD-E	Diykh et al., 2017	Establishing a weighted complex network with SVM classification	94.50
	Raghu et al., 2019	Analysis of Matrix Determinants with Multilayer Perceptron	96.85
	Proposed method	FHM + HDPAB+ ZIPMRM	93.48
		FHM + HDPAB+ LSTM	96.82
		FHM + HDPAB +BiLSTM	96.49
Schizophrenia dataset	Oh et al., 2019	11 layered CNN	98.07 and 81.26
	Prabhakar et al., 2020a	Nature inspired learning with machine learning	98.77
	Prabhakar et al., 2020b	Swarm intelligence with machine learning	92.17
	Proposed method	FHM + HDPAB+ ZIPMRM	94.83
		FHM + HDPAB+ LSTM	97.49
		FHM + HDPAB +BiLSTM	98.35

It is quite evident from Table 3 that the proposed methods produced a very good result in comparison with the previous results. Although at some places, the obtained classification accuracy may be slightly less by a range of 1–2%, but it should not deter the researchers and readers into concluding that the method is not versatile and innovative or even considering the necessity of the proposed method. In the field of machine learning and deep learning, every technique is pretty useful and has its own merits and demerits, and every proposed methodology has to be acknowledged unless it performs very worse. On considering this aspect, the proposed methodology performed very well by producing an overall high classification accuracy of 98.79% for epileptic dataset and 98.35% for the schizophrenia dataset. Moreover, previous methods have only concentrated on experimental analysis without any strong mathematical model to support it; however in our study, a good mathematical justification is also given, and it can be implemented to other biosignal processing datasets to obtain a good accuracy.

## Conclusion and Future Studies

In various fields of biomedical engineering, neural engineering, and neuroscience, the most widely used technique is EEG. Due to its low financial cost, very high temporal resolution accompanied by other exemplary attributes, it is widely used in different fields such as seizure detection, dementia analysis, alcoholism detection, analysis of sleep disorders, etc. To rely less on trained professionals, the efficient EEG signal classification is quite important. To the EEG data, a variety of conventional and advanced pattern recognition, soft computing, and machine learning algorithms were implemented. As the standardized EEG classification model of EEG includes preprocessing for removal of artifacts, feature extraction, feature selection, and classification, in our study, a fusion hybrid model called FHM was developed initially for feature extraction. A hybrid swarm algorithm called HDPAB was developed was feature selection and was followed by classification with ZIPMRM, deep learning, and eight other conventional classifiers. The best results are obtained for the A-E dataset wherein highest classification accuracy of 98.79% is obtained with Bi-LSTM, and for schizophrenia dataset, the highest classification accuracy of 98.35% is obtained. The second best results were obtained utilizing Bi-LSTM for D-E dataset reporting a classification accuracy of 98.75%, and for schizophrenia dataset, a higher classification accuracy of 97.49% is obtained when utilized with LSTM. The third best results were obtained when Bi-LSTM is utilized for C-E dataset reporting a classification accuracy of 98.66%, and for schizophrenia dataset, a classification accuracy of 95.67% is obtained with SVM. Future studies aim to develop efficient hybrid models and clubbing it with a variety of other deep learning techniques for the efficient classification of biosignals.

## Data Availability Statement

The relevant programming codes of this work can be obtained from the corresponding author upon request.

## Author Contributions

SP: visualization and experimentation. HR: experimentation and draft manuscript. CK: experimentation and critical analysis. D-OW: draft manuscript, writing and correction, and funding.

## Conflict of Interest

The author declares that the research was conducted in the absence of any commercial or financial relationships that could be construed as a potential conflict of interest.

## Publisher’s Note

All claims expressed in this article are solely those of the authors and do not necessarily represent those of their affiliated organizations, or those of the publisher, the editors and the reviewers. Any product that may be evaluated in this article, or claim that may be made by its manufacturer, is not guaranteed or endorsed by the publisher.
